# Breast-Contour-Preserving Procedure as a Multidisciplinary Parameter of Esthetic Outcome in Breast Cancer Treatment in The Netherlands

**DOI:** 10.1245/s10434-019-07265-3

**Published:** 2019-03-04

**Authors:** Annnelotte van Bommel, Pauline Spronk, Marc Mureau, Sabine Siesling, Carolien Smorenburg, Rob Tollenaar, Marie-Jeanne Vrancken Peeters, Thijs van Dalen

**Affiliations:** 1 Department of Surgery, Leiden University Medical Centre, Leiden, The Netherlands; 2 Dutch Institute for Clinical Auditing, Leiden, The Netherlands; 3 Department of Plastic and Reconstructive Surgery, Erasmus MC Cancer Institute, University Medical Centre Rotterdam, Rotterdam, The Netherlands; 4 Department of Research, Netherlands Comprehensive Cancer Organization (IKNL), Utrecht, The Netherlands; 5 Department of Health Technology and Services Research, MIRA Institute for Biomedical Technology and Technical Medicine, University of Twente, Enschede, The Netherlands; 6 Department of Medical Oncology, Netherlands Cancer Institute, Amsterdam, The Netherlands; 7 Department of Surgery, Netherlands Cancer Institute, Amsterdam, The Netherlands; 8 Department of Surgery, Diakonessenhuis Utrecht, Utrecht, The Netherlands

## Abstract

**Background:**

The rate of breast-conserving surgery (BCS) is used as an esthetic outcome parameter, while other treatments contribute also, such as neoadjuvant chemotherapy (NAC) enabling BCS or immediate breast reconstruction (IBR). This study explores these efforts to preserve the patient’s breast contour.

**Patients and Methods:**

All patients who underwent surgery for invasive breast cancer in The Netherlands between January 2011 and December 2015 were selected from the Dutch national breast cancer audit (*n* = 61,309). The breast-contour-preserving procedures (BCPP) rate was defined as the rate of primary BCS, BCS after NAC, or mastectomy with IBR. BCPP rates were calculated and compared by year of diagnosis, age categories, and individual hospitals.

**Results:**

The rate of primary BCS remained stable (53%) while the BCPP rate increased from 63% in 2011 to 71% in 2015 due to an increase in patients receiving BCS after NAC and mastectomy with IBR. Primary BCS rates increased with age (from 17% in patients aged < 30 years to 63% in patients aged 60–69 years), while the proportion of patients undergoing mastectomy with IBR decreased from 44% in patients < 30 years to 1% in patients ≥ 70 years. The BCPP rate was similar for all age groups except for patients > 70 years. BCPP rates varied between the different hospitals in The Netherlands, ranging from 47 to 88%.

**Conclusions:**

The chance of preserving the breast contour for patients with breast cancer has increased substantially over recent years. BCPP provides a comprehensive parameter of esthetic outcome of breast cancer surgery.

The quality of breast cancer treatment has received considerable attention in recent years. Identification of parameters that represent quality of breast cancer care is challenging. As survival rates for patients with primary breast cancer have improved considerably over the recent decades^1^ and local recurrence rates have decreased significantly,^2^ more effort is being directed to improve esthetic outcomes, reflecting an important aspect of quality of life. Previously, the proportion of patients undergoing breast-conserving surgery (BCS) has been used as a parameter reflecting esthetic outcome in breast cancer treatment. Recent population-based studies report stable BCS rates over the past years of approximately 60%,^3^,^4^ suggesting that esthetic outcomes of local treatment may not have improved over recent years.

Nonsurgical treatment modalities contribute to local esthetic outcome as well. The use of neoadjuvant systemic therapy influences the ability to perform BCS.^5^,^6^ Moreover, immediate breast reconstruction following mastectomy (IBR) or delayed breast reconstruction may also lead to desirable esthetic outcomes. Both neoadjuvant chemotherapy (NAC) and IBR are increasingly being used,^4^ and institutional preferences regarding the use of the former and surgical expertise with the latter have an impact on the surgical choice for BCS or mastectomy.

A parameter that comprises the combined efforts to preserve the breast contour may therefore be more appropriate to evaluate local esthetic outcome in breast cancer treatment. For this purpose, we defined “breast-contour-preserving procedure (BCPP)” as a parameter that encompasses all strategies to preserve the contour of the breast (primary BCS, BCS after NAC, and mastectomy with IBR). Within the NABON Breast Cancer Audit (NBCA),^3^ we explored BCPP as a local outcome parameter by evaluating trends over time in relation to age, and compared the frequencies of BCPP with primary BCS rates.

## Patients and Methods

### Data Source

Demographic and clinicopathological patient characteristics (age, histological subtype, grade, tumor–node–metastasis (TNM) classification) together with comprehensive multidisciplinary treatment information (surgical and medical adjuvant and neoadjuvant therapy) were collected prospectively for all newly diagnosed Dutch patients with breast cancer in the NABON Breast Cancer Audit (NBCA) since 2011.^4^ Registration was done by registrars of the Netherlands Cancer Registry and personnel of the individual hospitals. Patients receiving primary systemic treatment without subsequent surgical treatment were not registered in the NBCA. All female patients with primary invasive breast cancer without distant metastases diagnosed between January 1, 2011 and December 31, 2015 were extracted from the NBCA.

### Categories/Definitions

The surgical procedure was categorized as BCS or mastectomy as determined by the final operative procedure for the primary tumor. Patients who underwent BCS with subsequent mastectomy as a second or third operative procedure were categorized as having had a mastectomy. Patients who had undergone a mastectomy were subdivided by receipt of IBR. Of patients who had undergone BCS, those who had received NAC were identified and categorized as such. The endpoint of interest was BCPP, which was the final outcome of local treatment obtained by one of the following treatment strategies: (1) primary BCS, (2) BCS after NAC, and (3) mastectomy followed by IBR. The remaining patients underwent a mastectomy either primary or following NAC.

### Analysis

Descriptive statistics were used to describe the baseline characteristics of the study population. The proportions of patients who had undergone primary BCS were addressed for the study period of 5 years, and the effect of age on the rate of primary BCS was evaluated, as well as the variation in these proportions between individual hospitals. Similarly, the proportions within the categories that constituted the group of patients who had undergone BCPP were assessed and evaluated over time and in relation to age. Time trends of the rate of patients who had received primary BCS were compared with BCPP. All analyses were performed using SPSS 20 (IBM-SPSS Inc., Chicago).

## Results

During the study period, 61,309 patients were diagnosed and surgically treated for primary invasive breast cancer in 89 Dutch hospitals. Patient and tumor characteristics are summarized in Table 1. The median age of the patients was 61 years, and 74% of the patients were younger than 70 years old. The majority of patients were diagnosed with invasive ductal carcinoma (81%), and most tumors were staged as T1–2 (88%) and N0 (82%).

**Table 1:** Patient and tumor characteristics of 61,309 patients with invasive breast cancer in 2011–2015

	*n* (61,309)	%
Age (years)		
Below 30	305	1
30–39	2291	4
40–49	9139	15
50–59	16,058	26
60–69	17,788	29
70 or above	15,708	26
Histological subtype		
Ductal	49,677	81
Lobular	6936	11
Combination of ductal and lobular	1601	3
Other or unknown	3095	5
Grade		
I	14,233	23
II	26,340	43
III	15,431	25
Unknown	5305	9
Clinical tumor stage		
cTx	1946	3
cT0	72	0
cTis	1488	2
cT1	35,495	58
cT2	18,304	30
cT3	2943	5
cT4	1061	2
Clinical nodal stage		
cNx	1582	3
cN0	50,142	82
cN1	8697	14
cN2	323	1
cN3	565	1
Receptor type		
HR positive, HER2 negative	43,280	71
HR positive, HER2 positive	5006	8
HR negative, HER2 positive	2400	4
Triple negative	6498	11
Unknown	4125	7

*HR* hormone receptor; *HER2* human epidermal growth factor receptor 2

The frequencies of the treatment strategies leading to preservation of the breast contour are listed in Table 2. In 67% of all patients, the breast contour was preserved (BCPP): 53% of all patients (*n* = 32,520) underwent BCS as the primary and definitive surgical treatment, 5% had BCS following NAC (*n* = 3328), and 8% (*n* = 5023) of all patients underwent mastectomy combined with IBR. Patients who had received NAC accounted for one-tenth of all patients who had undergone BCS, while one-fifth of patients undergoing a mastectomy received IBR. Chemotherapy was administered to 41% of all patients: 5% of patients received NAC and subsequently underwent BCS, 7% of the patients received NAC and subsequently had a mastectomy, while 29% of patients received adjuvant chemotherapy.

**Table 2:** Surgical treatment strategies for patients diagnosed with invasive breast cancer, separated by year of diagnosis and age group, and hospital differences

	BCS		BCS		Mastectomy		**BCPP**	Mastectomy	
	NAC–		NAC+		IBR+			IBR–	
Total	32,520	53%	3328	5%	5023	8%	**67%**	20,438	33%
Year of diagnosis									
2011	5699	54%	367	3%	682	6%	**63%**	3905	37%
2012	7283	54%	501	4%	920	7%	**64%**	4801	36%
2013	7152	53%	748	6%	1102	8%	**67%**	4525	34%
2014	7308	53%	957	7%	1286	9%	**69%**	4377	31%
2015	5078	52%	755	8%	1033	11%	**71%**	2830	29%
Age group (years)									
Below 30	52	17%	39	13%	133	44%	**73%**	81	27%
30–39	619	27%	311	14%	593	26%	**67%**	768	34%
40–49	3522	39%	1084	12%	1566	17%	**68%**	2967	33%
50–59	9107	57%	1147	7%	1715	11%	**75%**	4089	26%
60–69	11,281	63%	662	4%	839	5%	**72%**	5006	28%
70 or above	7931	51%	83	1%	175	1%	**52%**	7519	48%
Hospitals									
Mean.	n.a.	53%	n.a.	5%	n.a.	8%	**67%**	n.a.	33%
Min.	n.a.	34%	n.a.	0%	n.a.	0%	**47%**	n.a.	12%
Max.	n.a.	67%	n.a.	21%	n.a.	28%	**88%**	n.a.	53%

*NAC* Neoadjuvant chemotherapy, *IBR* immediate breast reconstruction, *BCS* breast-conserving surgery, *BCPP* breast-contour-preserving procedure, *n.a.* not applicable

### Trends Over Time

**Fig. 1:**
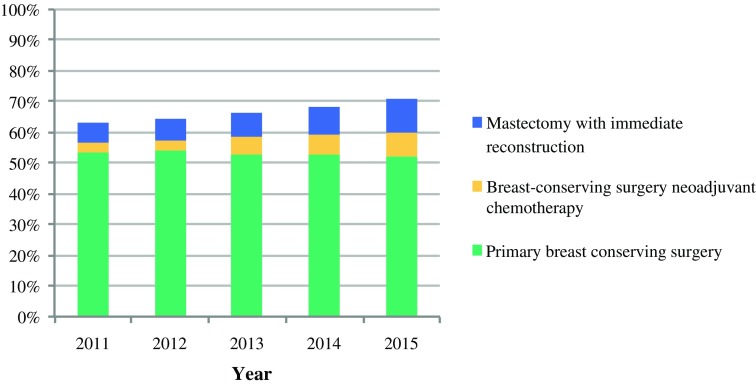
Annual proportion of patients who underwent a breast-contour-preserving procedure (BCPP) separated by multiple treatment modalities (2011–2015)

During 2011–2015, use of BCS following NAC and mastectomy with IBR both increased, from 3 to 8% and 6 to 11% of all patients, respectively. As a result, the overall frequency of BCPP increased significantly, from 63% in 2011 to 71% in 2015 (*P* < 0.001; Fig. 1; Table 2), and the proportion of patients who underwent a mastectomy without reconstruction decreased from 37 to 29%, i.e., a relative reduction of 22%. The proportion of patients undergoing mere BCS for invasive cancer in The Netherlands remained stable during the study period. A gradual increase was observed in the overall use of NAC, from 8% in 2011 to 16% in 2015.

### Age-Specific Frequency of BCS and BCPP

**Fig. 2:**
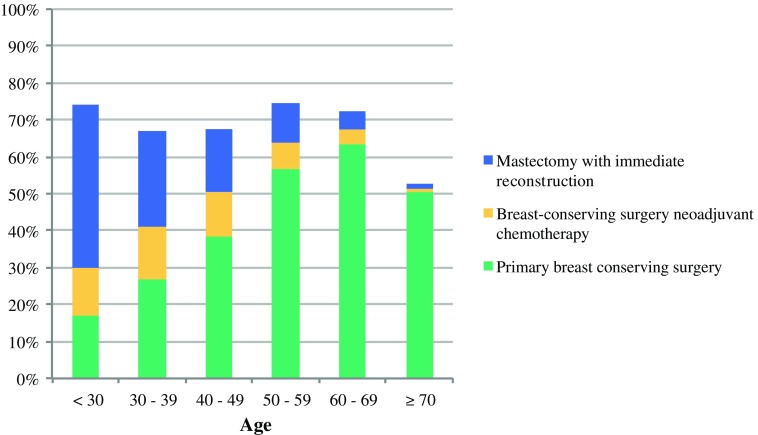
Multiple treatment modalities of the breast-contour-preserving procedure (BCPP) parameter for patients diagnosed with breast cancer separated by age

Table 2 presents the frequencies of the treatment strategies per age group. The overall frequency of BCPP was similar (approximately 70%) for all age categories, except for patients ≥ 70 years old (52%). The means used to preserve the breast contour varied per age group. The proportion of patients who underwent primary BCS was lowest under 30 years (17%) and highest (63%) in patients aged 60–69 years. With increasing age, both BCS after NAC and mastectomy with IBR rates decreased. Above the age of 70 years, a substantially lower percentage of primary BCS was observed (51%), and only a very low percentage of BCS after NAC (1%) and IBR (1%). Almost half of the oldest patients underwent a primary mastectomy. Figure 2 shows the cumulative age-specific proportions of the three treatment strategies to preserve the breast contour.

### Variation Between Hospitals

**Fig. 3:**
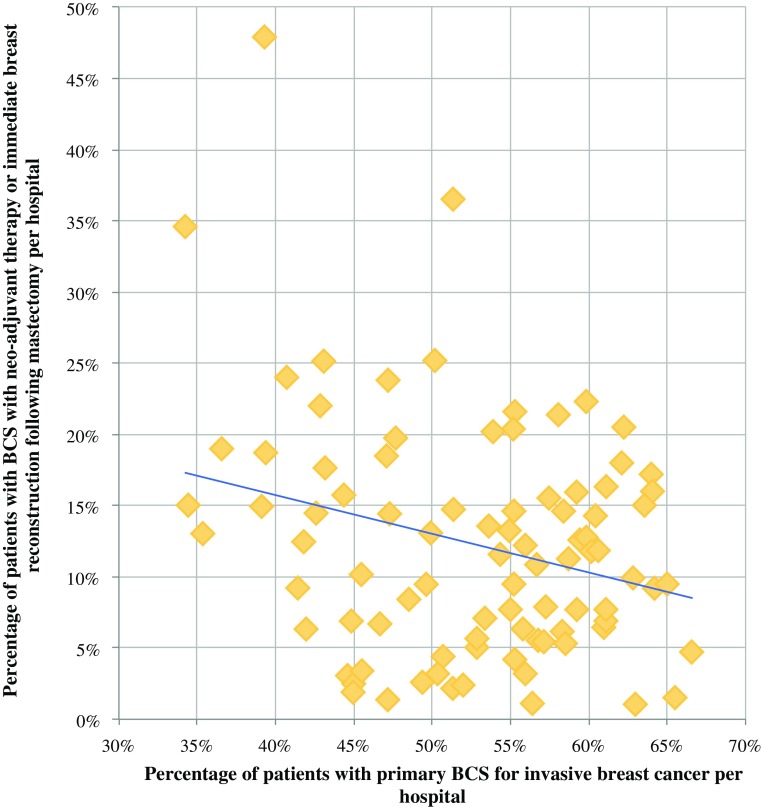
Correlation per hospital between the proportion of patients who underwent primary breast-conserving surgery (BCS) and the cumulative proportion of patients who had BCS following neoadjuvant chemotherapy and patients who underwent immediate breast reconstruction following mastectomy

The proportion of patients undergoing BCPP varied extensively between individual hospitals, and this range of BCPP (47–88%) was wider than the observed variation of BCS (37–67%). All three treatment strategies constituting BCPP showed a wide variation between hospitals (Table 2). There was an inverse relationship between the proportion of primary BCS and the other two strategies to preserve the breast contour per hospital (Fig. 3). The rates of BCS after NAC and mastectomy combined with IBR varied largely between hospitals: some hospitals never used BCS after NAC nor mastectomy with IBR, while other institutions performed BCS after NAC in up to 21% and IBR in up to 28% of patients. Hospital volume did not influence the institutional BCPP rate (data not shown).

## Discussion

We present BCPP as an esthetic local outcome measure in breast cancer patients. BCPP provides a comprehensive parameter encompassing various treatment strategies to maintain the breast contour in patients treated for breast cancer. While in The Netherlands the rate of BCS remained stable during the study period, the rate of BCPP increased, from 63% in 2011 to 71% in 2015. This increase is the result of increased use of BCS after NAC and mastectomy with IBR.

To the best of the authors’ knowledge, no other studies have described BCPP as a composite measure to evaluate local esthetic outcome. Many studies have reported trends of the separate surgical, reconstructive, and medical modalities in patients treated for primary breast cancer.^4^,^7^–^11^ Population-based BCS rates have remained stable in recent years in Brazil^7^ and The Netherlands,^4^ while an increase was observed in some other European countries.^11^ Over a similar time period, a decrease in the proportion of patients undergoing BCS was seen in the USA (from 66.6% in 1998 to 61.9% in 2011).^8^,^12^–^15^ Other studies have reported significant institutional and regional differences in BCS rates, ranging from 20 to 84%.^11^,^16^–^20^ Increased use of mastectomy combined with IBR over time, differences in IBR rates between countries,^4^,^7^,^8^,^21^–^24^ as well as more frequent application of NAC have also been reported.^4^,^5^,^25^–^28^ The observed rise in the rate of BCPP in relation to the observed stable primary BCS rate demonstrates that the composite endpoint has additional value as a local esthetic outcome parameter. This is illustrated in the present study, since a stable rate of primary BCS masks a 22% proportional decrease of patients who underwent a plain mastectomy.

The BCPP rate was similar for most age groups, but the strategies used to maintain the breast contour varied largely between the different age groups. Primary BCS was increasingly used when patients were older, and a concomitant decrease was observed for the proportions of patients who underwent BCS after NAC and those who underwent mastectomy with IBR. In the very young age group, IBR accounted for half of the patients in whom the breast contour was preserved. The difference in the proportion of patients who had primary BCS in relation to the overall proportion undergoing BCPP (17% and 73%, respectively) was most profound in these very young patients (< 30 years old). This is in part explained by previous guidelines advising against BCS in the young because of the higher risk of local recurrence and diagnosed genetic mutations.^29^

In patients aged > 70 years, the low rate of BCPP merely reflected the rate of BCS, since BCS after NAC and mastectomy with IBR were infrequently used (1% and 1%, respectively). The absence of evidence in support of adjuvant chemotherapy in patients older than 70 years explains why NAC was hardly ever administered. The low rate of mastectomy with IBR seems conceivable too, although the extent to which patient preferences explain the observed higher mastectomy rate remains unanswered. BCPP as such was of little additional value in these elderly patients.

The rate of BCS has been promulgated as a quality indicator.^30^ When performing primary BCS, a delicate balance exists between the esthetic and oncological aims of the surgery: a wider excision may lead to a worse esthetic result, while too narrow an excision may leave residual tumor tissue. Striving for a high BCS rate may unintentionally lead to the perverse incentive of aiming for the lowest possible positive margin rates by resecting larger amounts of breast tissue. BCPP serves the aim of measuring esthetic outcome more appropriately, as it appreciates at least the combined efforts and different treatment strategies to maintain the shape of the breast, which is in itself a desirable esthetic outcome.

While BCPP more or less annihilated conventional age-specific BCS rates, no such effect was observed for institutional differences. Despite an apparent interplay between the various strategies used to preserve the breast contour (illustrated by the observed inverse association between the rate of BCS and the proportion of patients who underwent BCPP), the net effect of the hospital variation in BCS after NAC and mastectomy with IBR resulted in an observed wider range of the proportion of BCPP than the hospital variation in BCS rates. Previous studies using data from the NBCA studied the variation of NAC rates^25^ and the proportion of patients undergoing mastectomy combined with IBR.^21^,^31^ Patient and tumor characteristics and hospital factors did account for institutional variation, but the number of treated patients per hospital was not a factor associated with higher rates of NAC or IBR. In another study, we also observed that surgeons’ and plastic surgeons’ preferences had an impact on the institutional IBR rate.^32^ Much of the observed institutional variation remains unexplained. Several hospitals in the present study that never applied NAC or provided IBR might explain the wider range of BCPP rates. As these hospitals had no means other than primary BCS to enhance their BCPP rate, these institutions fell behind as others were improving their BCPP rate. Obviously, this hypothesis urges the need for additional in-depth analysis of the observed institutional variation.

Having a national multidisciplinary audit for breast cancer care enabled us to analyze questions with large numbers of patients. This is a strength of the present study, and the population-based data are also suitable to study time trends. The absence of information regarding important patient characteristics such as smoking status and body mass index is a limitation of the NBCA. These factors may well affect the eligibility of patients to undergo immediate breast reconstruction. Moreover, the lack of data about delayed reconstruction may limit the interpretation of results since to some extent. In addition, institutional availability and use of oncoplastic surgical techniques as well as radiotherapy indications have an impact on the desirability to perform BCS or prosthesis use, respectively. However, data regarding the use of oncoplastic techniques lacked sufficient detail to take into consideration. Referral patterns between hospitals, e.g., patients who underwent surgery at an institution another than the hospital where NAC was administered, could not be addressed. Finally, information regarding the achieved and perceived success of BCS as well as of an immediate breast reconstruction was not available, but would importantly enhance the value of BCPP as an outcome parameter.

BCPP provides insight into the various ways in which breast cancer patients can retain their breast contour, and the result reflects combined multidisciplinary efforts. Although it still lacks information about the perceived esthetic outcome, BCPP is an important step in providing more information than the rate of BCS alone. Achievement of a 100% preservation score is not considered to be an ultimate goal. We acknowledge that multiple factors influence the treatment options that can and will be offered to patients, and the patient’s decision. Notwithstanding these limitations, this study supports the use of the BCPP rate as a local outcome parameter, and an institutional BCPP rate of 75% in patients younger than 70 years may well be defined as an appropriate norm value for good esthetic outcome of local treatment.

## Conclusions

BCPP as a composite parameter provides insight into and understanding of the preservation of the breast contour in primary breast cancer patients, appreciating the various ways to maintain the contour of the breast. This study demonstrates that, while the BCS rate remained stable over recent years, the proportion of patients in whom the breast contour was preserved increased while the proportion who underwent a plain mastectomy decreased by one-fifth. At the same time, unexplained institutional differences in the BCS rate persist when applying the rate of BCPP as a quality indicator, and this should motivate future research.
